# Sternal lymphadenopathy in dogs with malignancy in different localizations: A CT retrospective study of 60 cases

**DOI:** 10.3389/fvets.2022.1019196

**Published:** 2022-10-21

**Authors:** Alessia Cordella, Jimmy Saunders, Emmelie Stock

**Affiliations:** Department of Medical Imaging of Faculty of Veterinary Medicine, Ghent University, Merelbeke, Belgium

**Keywords:** sternal lymph node, computed tomography, thorax, neoplasia, tumor

## Abstract

Sternal lymph nodes (SLNs) drain a multitude of regions in dogs, including the pectoral and shoulder region, the thoracic wall and mammary glands, the mediastinum, thymus, diaphragm, and the ventral abdominal wall and peritoneal cavity. Neoplastic conditions of these regions can lead to sternal lymphadenopathy. The aim of this study was to assess the most frequent localizations of the primary neoplasia and the most frequent tumor types in dogs with sternal lymphadenopathy. Computed tomographic (CT) characteristics of SLNs in dogs with confirmed neoplasia were also described. For this single-center retrospective descriptive study, dogs with sternal lymphadenopathy and cytological or histological diagnosis of neoplasia were included. Sixty dogs fulfilled the inclusion criteria: 30 (50%) with thoracic neoplasia, 19 (32%) with abdominal neoplasia, 6 (10%) with neoplasia of the front limbs and 5 (8%) with generalized neoplasia. Based on the cytological/histological diagnosis of the primary neoplasia, 31/60 (52%) dogs presented with a sarcoma, 15/60 (25%) with carcinoma, and 14/60 (23%) with round cell tumor. The presence of heterogeneous contrast enhancement was more frequent in dogs with sarcoma, while the concomitant presence of other abnormal lymph nodes was more frequent in dogs with round cell neoplasia. Tumors of different types and in different location can result in sternal lymphadenopathy in dogs. The most frequent in this study were thoracic and abdominal neoplasia, followed by neoplasia of the shoulder region. Sarcoma was the most common tumor type detected in this study, and the main CT characteristic of the SLNs in case of sarcoma was heterogeneous contrast enhancement.

## Introduction

Sternal lymph nodes (SLNs) are present in variable number in the dog, and they are the only lymph nodes of the ventral thoracic lymph center (1). In dogs, unlike other species, only the cranial SNLs are present, and they are located dorsal to the sternum, usually at the level of the second intercostal space, ventral to the internal thoracic artery and vein (1). The afferent lymphatic vessels to the SLNs are coming from the pectoral and shoulder region, from the ventral thoracic wall and mammary glands (three most cranial pairs of glands), from the mediastinum, thymus, diaphragm, and from the ventral abdominal wall and peritoneal cavity (1–3).

Normal SLNs are reported to be not apparent on radiographic images, but enlarged SLNs appears as a radiopaque mass dorsal to the second sternebra, and is more easily evaluated on lateral radiographs (4, 5). Due to the lack of superimposition and the greater contrast resolution compared to radiography, computed tomography (CT) is a useful modality for the assessment of thoracic lymph nodes in dogs (6–8). The normal CT appearance of the SLNs has been previously reported in dogs: the shape of the lymph nodes was described as rounded or ovoid (7), and the dimensions of the SLNs showed a positive correlation with body weight in previous studies (6, 8). To assess the SLN enlargement, a ratio with the dorso-ventral diameter of the second sternebra has been suggested in previous studies (6, 8). An increased ratio, together with increased pre-contrast attenuation of the SLNs has proved to be useful in the diagnosis of metastatic lymphadenopathy in dogs (9).

The SLNs can be enlarged due to a variety of conditions (neoplastic, inflammatory and hematologic) as described in previous study about radiographic appearance of the SLNs in dogs and cats (4). According to this study, the most common disease associated with sternal lymphadenopathy is lymphoma, followed by splenic hemangiosarcoma (4).

The aims of the current study were: (1) to assess the localizations of the primary neoplasia in dogs with sternal lymphadenopathy, (2) to assess the more frequent tumor types (3) to describe the CT characteristics of SLNs in dogs with confirmed neoplasia, and (4) to determine which CT characteristics of the SLNs can help distinguish the different tumor types.

## Materials and methods

### Case selection criteria and study design

For this single-center retrospective descriptive study, the electronic medical records of all dogs that had undergone CT examination at Ghent University between May 2016 and May 2022 were reviewed. Eligibility criteria were as follows: (1) presence of keywords “sternal lymph node, sternal lymphadenopathy, sternal lymphadenomegaly” in the CT report (2) pre- and post-contrast CT examination of the whole body available for review (3) cytological or histological diagnosis of neoplasia (4) data regarding age, sex, breed and bodyweight available. All selected CT examination were reviewed, and cases in which SLNs were considered normal were excluded from the study. Non-diagnostic or unavailable cytological or histological evaluation and incomplete CT studies represented additional exclusion criteria.

All imaging procedures were performed solely for patients' benefit and for standard diagnostic and monitoring purposes. Previous informed written consent was obtained from all dog owners. All the procedures performed complied with the European legislation “on the protection of animals used for scientific purposes” (Directive 2010/63/EU).

Patients included were then divided in four groups based on the localization of the diagnosed neoplasia: thoracic neoplasia, abdominal neoplasia, front limb neoplasia, and generalized neoplasia. Patients in the thoracic group were then subsequently divided in four sub-groups: pulmonary neoplasia, mediastinal neoplasia, thoracic wall neoplasia, and cardiac neoplasia.

Based on the cyto/histological diagnosis of the primary neoplasia, cases were divided in: round cell tumors, carcinomas and sarcomas.

### CT scanning techniques

Computed tomographic data were obtained with a 4-row MDCT unit (Lightspeed Qx/I, General Electric Medical Systems) or with a 320-row MDCT unit (Aquilion One, Toshiba Medical Systems, Otawara, Japan). Technical parameters were as follows: for the 4-row unit: helical modality, 120 kVp, 140 mAs, image matrix 512 × 512, 1.25 mm slice thickness; for the 320-row unit: helical modality, 120 kVp, 200 mAs, image matrix 512 × 512, 0.5 mm slice thickness.

Dogs were all scanned in sternal recumbency on the CT table, with the head first, front limbs cranially extended and hindlimbs caudally extended. For all studies, a pre-contrast series of the whole body was acquired, followed by at least one post-contrast series acquired between 2 and 5 minutes after injection of iodinated contrast agent (iohexol 370 mgI/mL, 2 mLI/kg dosage followed by a saline flush) *via* a cephalic vein either manually or with single/dual barrel injector system.

### CT image analyses and evaluation

CT images were retrieved from the PACS and analyzed using dedicate freestanding workstations (OsiriX v5.8.5 64-bit, Geneva, Switzerland) by a third-year European College of Veterinary Imaging resident (A.C.). Two-dimensional (2D) multiplanar reformations (MPRs), postprocessing techniques were used in all cases, and window settings were subjectively dynamically adjusted to obtain optimal visualization of the mediastinal structures. Number and dimensions of the sternal lymph nodes (SLNs) (measured as length, width and height) were assessed for all dogs. In addition, the SLNs/sternum ratio was calculated, as previously reported (6). When more than one SLN were present, the dimensions of the biggest one were measured. The shape of the SLNs was indicated as rounded, ovoid-shaped or irregular. The contrast enhancement was subjectively assessed as homogeneous or heterogeneous. The presence of pleural, pericardial or peritoneal effusion and concomitant lymphadenopathy (subjective enlargement) of other thoracic lymph nodes (cranial mediastinal and tracheobronchial) was also recorded.

### Statistical analyses

The normality of distribution of the data was evaluated based on visual inspection of histograms, the Shapiro-Wilk test and the Q-Q plots. Categorical variables were expressed as percentages, numerical variables as median and interquartile range (for data with non-normal distribution) and mean and standard deviation (for data with normal distribution). Differences among groups for continuous variables were evaluated using the Kruskall-Wallis test (for data with non-normal distribution) and one-way ANOVA test (for data with normal distribution) with Bonferroni's post hoc analysis. Frequencies of the CT variables evaluated in the study were compared among the study groups using Fisher's exact test. The free-software R (http://www.r-project.org/) was used for statistical analyses. The level of significance was set at *p* < 0.05.

## Results

### Animals

Based on the inclusion criteria, 60 dogs were included in the study. Median age of the dogs included was 8 (1-14) years, and median weight was 27, 5 (7-62) kg. Thirty-two out of 60 dogs were males (53%), of which 16 neutered, and the remainder 28/60 (47%) were females, of which 21 neutered. Six out of 60 (10%) were crossbreed dogs, and 34 different breeds were represented, including eight (14%) Bernese Mountain dogs, four (7%) Australian Shepherds, and three (5%) Beagles.

No statistically significant differences were present between groups (sarcoma, carcinoma and round cell neoplasia) regarding the sex, sexual status and breed. Dogs with round cell neoplasia were younger, with a mean age of 6.5 (± 1.74) years, compared to dogs with carcinoma (9.40 ± 2.59 years) (*p* = 0.0058) and sarcomas (8.39 ± 2.55 years) (*p* = 0.0533), however not significantly for the latter. Although not statistically significant (*p* = 0.138), the median bodyweight of the dogs included in the sarcoma group was higher (31 kg; 8–62) than the round cell (24.50 kg; 7–37) and carcinoma (18 kg; 9–40) groups.

When considering the tumor location (front limb, thorax, abdomen, and generalized neoplasia), no statistically significant difference in the signalment was found between groups.

### Localization and type of the primary neoplasia

**Figure 1:**
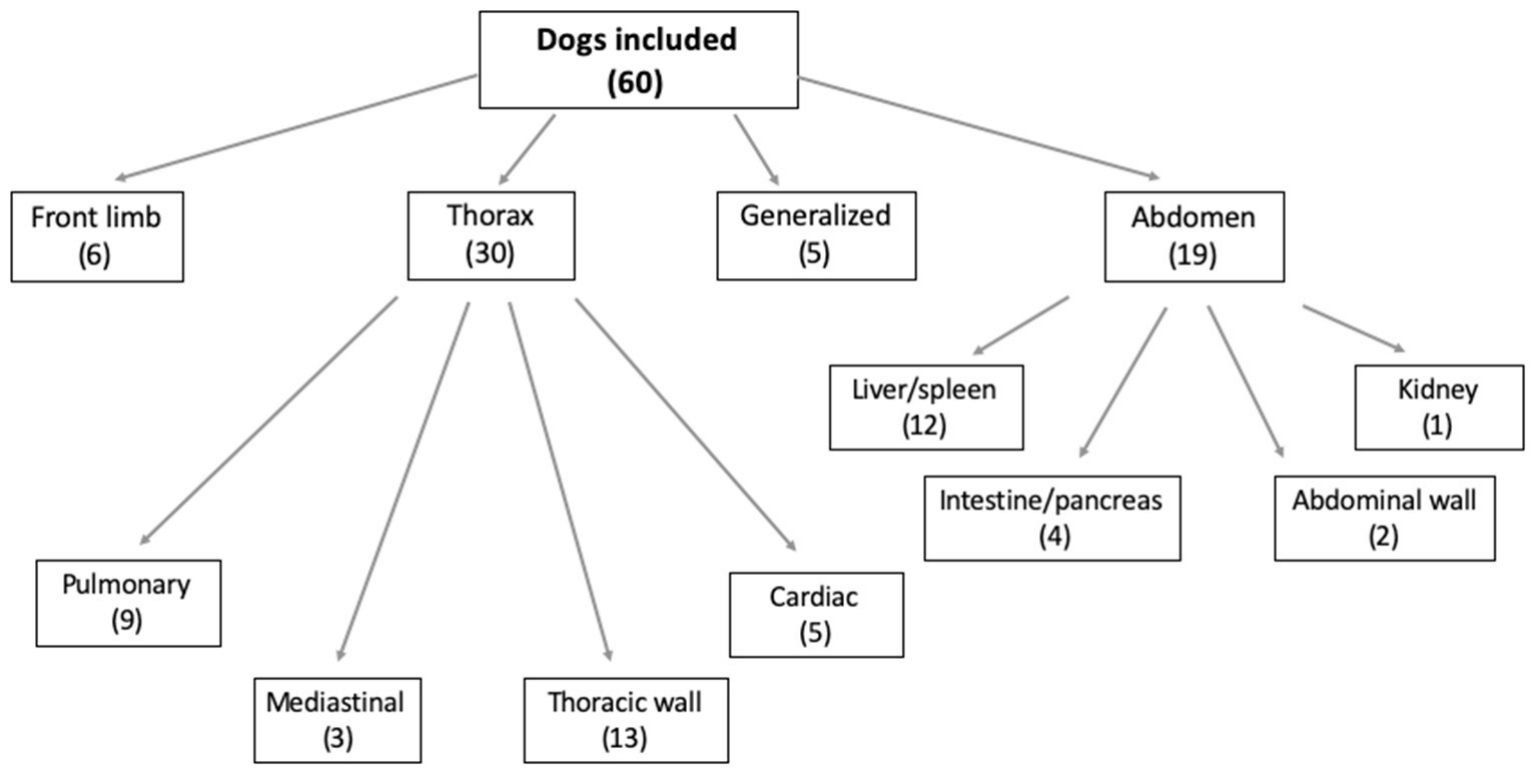
Chart illustrating the location of the primary neoplasia in the dogs included. The number of dogs included is in brackets.

Figure 1 illustrates the number of patients included in each study group, divided based on the localization of the primary neoplasia.

**Figure 2:**
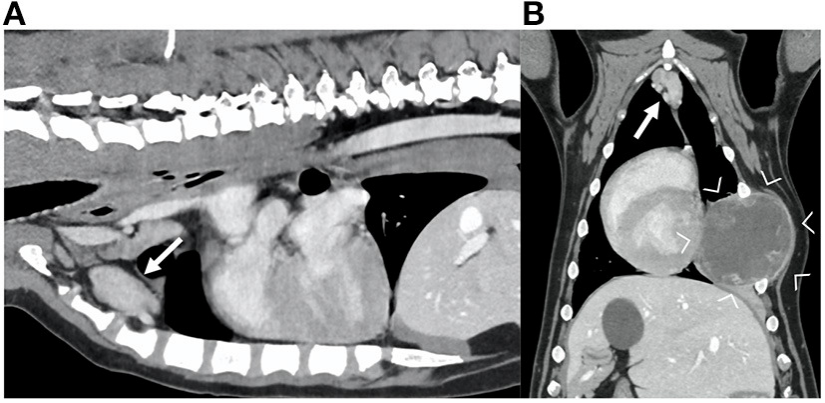
Sagittal **(A)** and dorsal **(B)** reconstruction of the post-contrast images of the thorax of a dog diagnosed with a hemangiosarcoma of the rib (between arrowheads). Note the markedly enlarged sternal lymph nodes (arrows), with irregular shape and showing heterogeneous contrast enhancement.

**Figure 3:**
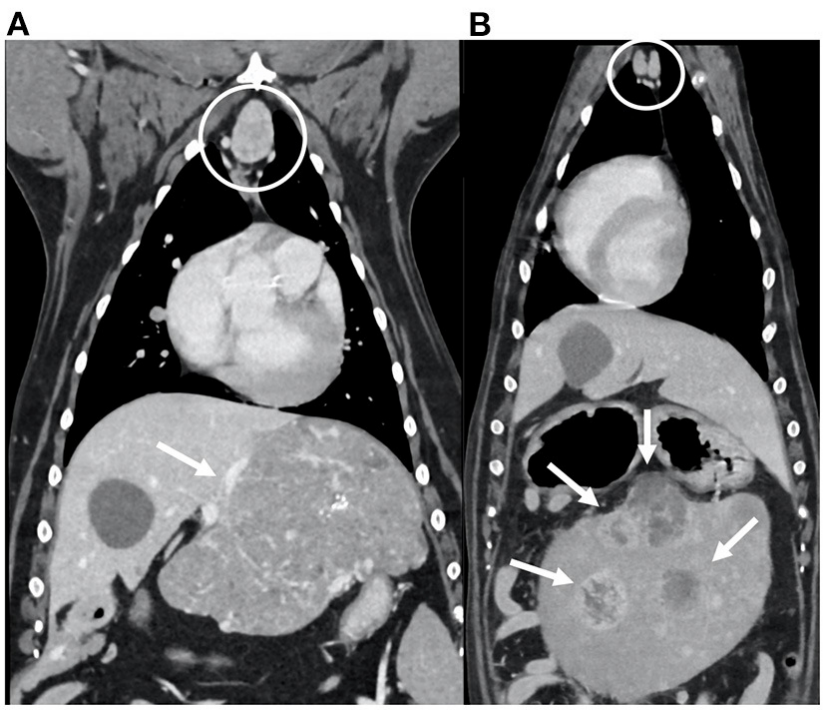
Dorsal reconstructions of the post-contrast images of the thorax and cranial abdomen of two dogs diagnosed with abdominal neoplasia (arrows): hepatic carcinoma **(A)** and splenic hemangiosarcoma **(B)**. Note the enlarged sternal lymph nodes (within the circles), with ovoid shape; the enhancement is heterogeneous in **(A)** and homogeneous in **(B)**.

**Figure 4:**
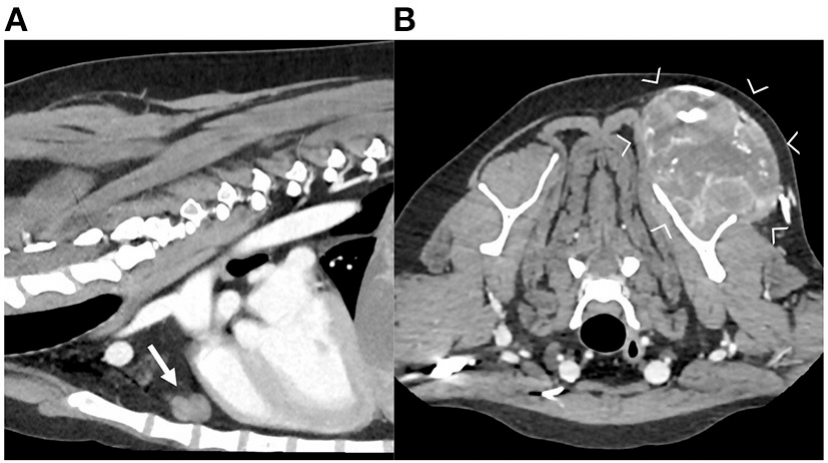
Sagittal **(A)** and transverse **(B)** reconstructions of the post-contrast images of a dog diagnosed with soft tissue sarcoma of the left front limb (between arrowheads). Note the enlarged sternal lymph node (arrow), with irregular shape and heterogeneous contrast enhancement.

Half of the included dogs presented a primary thoracic neoplasia (30/60; 50%); among these, the majority of the dogs were diagnosed with a thoracic wall neoplasia (Figure 2). Of the 19/60 (32%) dogs with diagnosed abdominal neoplasia, 12/19 (63%) presented with hepatic and/or splenic neoplasia (Figure 3). Some dogs presented with a neoplasia of the front limbs (6/60; 10%): all these dogs showed a mass arising from the scapula or from the soft tissues surrounding the scapula/shoulder region (Figure 4). The minority of the included dogs (5/60; 8%) presented generalized neoplasia, such as multicentric lymphoma.

Frequency of the included tumor types are summarized in Table 1.

**Table 1:** Anatomical location and tumor type of the 60 dogs included in the study.

**Anatomical location**	**Sarcoma (*n* = 31)**	**Carcinoma (*n* = 15)**	**Round cell neoplasia (*n* = 14)**
Front limb (6)	Soft tissue sarcoma (4); hemangiosarcoma (1)		Histiocytic sarcoma (1)
Thorax (30)	osteosarcoma (7); hemangiosarcoma (6); mesothelioma (4); chondrosarcoma (2)	Bronchoalveolar carcinoma (5); mammary gland adenocarcinoma (1); esophageal carcinoma (1); thymic carcinoma (1)	Pulmonary histiocytic sarcoma (2); spinal lymphoma (1)
Generalized (5)			Multicentric lymphoma (5)
Abdomen (19)	Hemangiosarcoma (7)	Hepatocellular carcinoma (4); jejunal/pancreatic adenocarcinoma (2); renal cell carcinoma (1)	Histiocytic sarcoma (2); high grade mast cell tumor (1); intestinal lymphoma (1); hepatic T-cell lymphoma (1)

### CT findings

**Figure 5:**
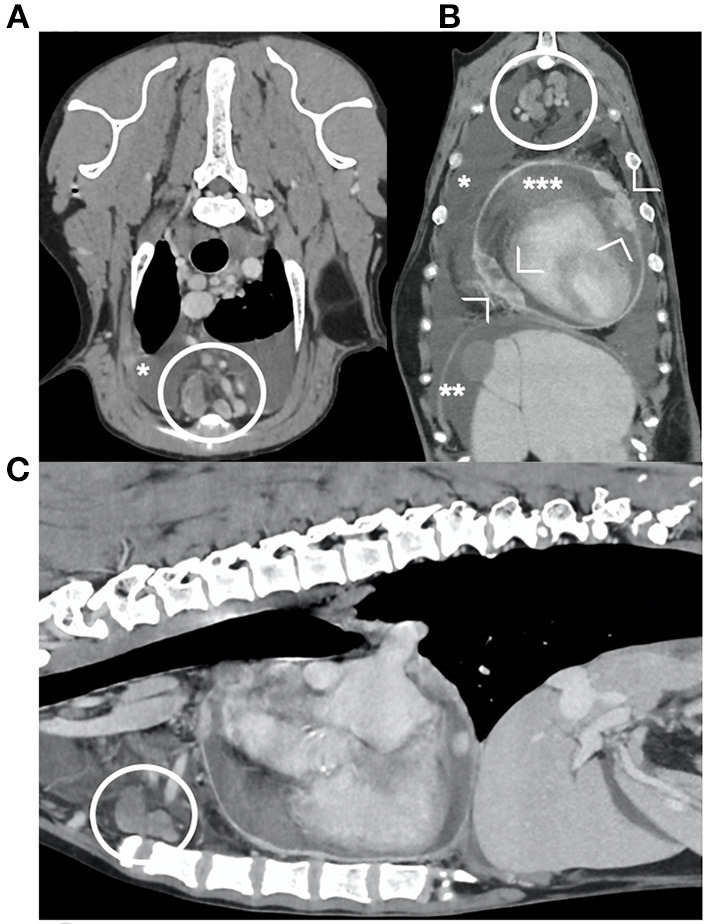
transverse **(A)**, dorsal **(B)** and sagittal **(C)** reconstructions of the post-contrast images of the thorax and cranial abdomen of one dog diagnosed with mesothelioma. Presence of pleural (*), peritoneal (**) and pericardial (***) effusion, and nodular thickening of the pericardium (arrowheads). Note the enlarged sternal lymph nodes (within the circles), with irregular shape and heterogeneous enhancement.

The majority of dogs presented with two SLNs (37/60; 62%) detected at CT examination, 14/60 (23%) had three SLNs visible and 9/60 (15%) had only one visible. Median length was 24 (10–39 mm), median width was 12,5 (8-30) mm and median height was 14 (8-35) mm. Median SLN/sternum ratio was 1.11 (0.78–2,79). Most of the dogs presented ovoid-shaped SLNs (42/60; 70%), 12/60 (20%) had irregularly shaped SLNs and 6/60 (10%) had rounded SLNs. The contrast enhancement was subjectively assessed as homogeneous in 36/60 (60%) dogs and heterogeneous in the remaining 24 (40%). Pleural effusion was present in 18/60 (30%) dogs, peritoneal effusion in 11/60 (18%) dogs and pericardial effusion in 5/60 (8%) dogs (Figure 5). Four out of the eleven dogs with peritoneal effusion presented with hemoabdomen due to ruptured mass.

In 15/60 dogs, other thoracic lymph nodes (cranial mediastinal and/or tracheobronchial) were also enlarged at CT examination.

No statistically significant difference was found when comparing the CT features of the SLNs between the different localizations of the primary neoplasia.

### Correlation of CT findings with tumor type

Based on the cytological/histological diagnosis of the primary neoplasia, 31/60 (52%) dogs presented with a sarcoma, 15/60 (25%) with carcinoma, and 14/60 (23%) with round cell tumor. The CT findings of the dogs divided in the three different groups (based on the tumor type) are summarized in Table 2. No statistically significant difference was noticed in the length, width or height of the largest sternal lymph node among the three groups. The presence of SLNs with heterogeneous contrast enhancement was significantly more frequent in dogs with sarcoma with respect to dogs with carcinoma (*p* = 0.011) and round cell tumor (*p* = 0.069), although the latter only close to statistical significance. The concomitant presence of other abnormal lymph nodes was significantly more frequent in dogs with round cell neoplasia compared to dogs with carcinoma (*p* = 0.007), while no statistical significance was noticed in the comparison between other groups.

**Table 2:** Comparison of CT findings in the three groups of dogs divided based on the cytological diagnosis in sarcoma, carcinoma and round cell neoplasia.

**CT findings**	**Sarcoma (*n* = 31)**	**Carcinoma (n = 15)**	**Round cell neoplasia (*n* = 14)**	***P-*value**
Length (mm)*	24,74 (6.47)	20,73 (7.18)	26,07 (7,97)	0.0988
Width (mm)§	12 (10–17.50; 8–30)	11 (10 −16;8–24)	13 (12-19; 10-24)	0.306
Height (mm)§	15 (12-20; 8-35)	11 (10-15; 9-22)	15,5 (12,5-19; 10-29)	0.05
SLN/sternum ratio§	1.08 (0.92–1.35;0,71–2.18)	1.19 (0.98–1.47; 0.79–1.84)	1.23 (1.00–1.49; 0.79–2.79)	0.654
Shape	Ovoid 22 (71%) Rounded 2 (6%) Irregular 7 (23%)	Ovoid 10 (7%) Rounded 3 (20%) Irregular 2 (13%)	Ovoid 10 (71%) Rounded 1 (7%) Irregular 3 (21%)	0.749
**Contrast enhancement**	Homogeneous 12 (39%) Heterogeneous 19 (61%)	Homogeneous 13 (86%) Heterogeneous 2 (14%)	Homogeneous 11 (79%) Heterogeneous 3 (21%)	0.00192
Effusion	Pleural 8 (26%) Peritoneal 6 (19%) Pericardial 5 (16%)	Pleural 5 (33%) Peritoneal 4 (13%) Pericardial 0	Pleural 2 (14%) Peritoneal 1 (7%) Pericardial 0	0.205
**Other LNs**	3 (10%)	5 (33%)	7 (50%)	0.001

Data are reported as frequency and percentage of total cases. *Data reported as average and standard deviation (SD); ^§^Data reported as median + interquartile range (IQR) and range. A *p* < 0.05 was considered significant (findings in bold).

## Discussion

The SLNs in dogs receive drainage from a variety of regions, including the thoracic wall (and also ribs and sternum), the shoulder girdle, the diaphragm, the mediastinum, the ventral abdominal wall, the abdominal cavity and the mammary gland complex (1, 3). Due to the large territory drained by the SLNs, sternal lymphadenopathy is a relatively common finding, especially in dogs with neoplasia (9). Previous reports described lymphoma as the most common cause of sternal lymphadenopathy in dogs (4, 9) and in one study sternal lymphadenopathy was the most common thoracic lesion observed in dogs with multicentric lymphoma (10). To date, despite the presence of some studies regarding the SLNs in dogs, little data are available regarding the most common location of neoplasia in dogs with sternal lymphadenopathy. In the present study, the most frequent location of the primary tumor in our population was the thorax (half of the included cases): of these 30 dogs with primary thoracic neoplasia, the majority presented with a neoplasia of the thoracic wall (sternum, ribs, vertebrae or soft tissues of the thoracic wall). Pulmonary neoplasia was the second most common location after thoracic wall. Other lymph nodes have been previously described to be involved in dogs with pulmonary neoplasia, in particular the tracheobronchial lymph nodes, that also carry a prognostic relevance (11–13). Involvement of the SLNs in case of pulmonary neoplasia, and in particular pulmonary carcinoma, similarly to our findings, was reported in few cases in previous studies (4, 9), but its significance in terms of survival rate or survival time have been to date not assessed.

The second most common location of the primary neoplasia in our study population was the abdomen. Among the 19 dogs presented with abdominal neoplasia, the majority had a hepatic and/or splenic neoplasia. This result is partially in accordance with previously reported study, in which splenic hemangiosarcoma was the most common abdominal neoplasia (second, after lymphoma) in dogs with radiographically detected sternal lymphadenopathy (4). In all cases included in the previous study, dogs presented with hemoabdomen, considered potentially the cause of the sternal lymphadenopathy (4). In our population, peritoneal effusion was present in only half of the cases with splenic or hepatic neoplasia, and in only two cases with splenic hemangiosarcoma the effusion was confirmed as hematic in origin. Splenic hemangiosarcoma associated with hemoabdomen was therefore a relatively uncommon finding in our population compared to previously reported. The presence of abdominal, pleural and pericardial effusion was different between the different tumor types, although not reaching statistical significance. While pleural effusion was more frequent in case of carcinoma, the five dogs with pericardial effusion were all included in the sarcoma group, four with mesothelioma and one with cardiac hemangiosarcoma. Mesothelioma is a rare neoplasm in dogs, and CT features usually include cavitary effusion and presence of pleural masses (14). In a previous case report of confirmed mesothelioma, thoracic lymphadenopathy was not present (14), while one dog with malignant mesothelioma included in a previous study presented with metastatic SLNs (9). Overall, the majority of dogs in all groups did not show cavitary effusion, suggesting that the presence of sternal lymphadenopathy is not correlated with the presence of effusion.

Surprisingly, in the current study, a relatively high number of dogs presented with neoplasia of the front limb. In particular, in all six cases included, the primary neoplastic mass was located at the level of the scapular/shoulder region. As known from anatomical studies (1–3), the shoulder region in dogs is drained through the SLNs; nevertheless, the presence of sternal lymphadenopathy in dogs with malignancy of this region has not been previously reported. This finding highlights the importance to include the SLNs while staging lesions of the shoulder region, together with the evaluation of other lymph centers more frequently assessed (such as cervical superficial and axillary).

Opposed to previously reported (4), in our population the sternal lymphadenopathy secondary to generalized neoplasia, such as multicentric lymphoma, was an uncommon finding. This result can be explained by the difference in the imaging modalities used in the two studies: CT is not routinely used for the staging of lymphoma at our institution, most likely leading to the low percentage of cases showing this type of neoplasia. On the contrary, in the study of Smith et al. the sternal lymphadenopathy was detected at radiographic examination (4). Similarly, the relatively lower percentage of splenic hemangiosarcoma in our population compared to previous study (4) can be related to the more frequent ultrasonographic diagnosis compared to CT evaluation in these cases. The CT examination is on the contrary necessary for more complex localization, as for example thoracic wall, lungs, and shoulders, with higher percentage of these tumors included in the current study.

The appearance of the SLNs was similar in dogs with different tumor types and location, but some differences were noticed between groups. As expected, multiple lymph nodes were found to be enlarged more frequently in dogs with round cell neoplasia compared to other tumor types. The contrast enhancement was significantly more frequently heterogeneous in dogs with sarcoma, compared in particular to dogs with carcinoma, where the contrast enhancement was more homogeneous. The heterogeneous contrast enhancement is a common CT feature of many sarcomas in dogs (15–18), and the contrast enhancement of the SLNs in our population likely reflects the contrast enhancement of the primary lesion. Unfortunately, the cytological and/or histological confirmation of metastatic lymphadenopathy was available in only one third of the patients included in the study. This is partially a limitation, as the primary aim of the study was to assess the frequency of the neoplasia and the localization of the primary neoplasia in dogs with sternal lymphadenopathy, despite the type of lymphadenopathy (metastatic or reactive). Furthermore, all dogs included in the study had a confirmed neoplasia in one of the regions drained by the sternal lymph node, and no signs of concomitant inflammatory condition. In addition, the size of the SLNs and the ratio with the sternum found in our population suggest a lymph node metastatic process according to previous study (9). Nevertheless, as the CT characteristics of metastatic and reactive lymph nodes show high degrees of overlap, CT findings alone cannot lead to a definitive diagnosis. For this reason, and for the relatively low number of cases included and the retrospective nature of the study, further studies are needed in order to confirm the clinical significance of the current findings.

In conclusion, many primary tumors in many different location can result in sternal lymphadenopathy in dogs. Together with thoracic and abdominal neoplasia (including tumors of the thoracic wall, lungs, heart and pericardium, and multiple abdominal organs), the presence of sternal lymphadenopathy can also be related to neoplasia of the shoulder region. Sarcoma was the most common tumor type detected in this study, and the main CT characteristic of the SLNs in case of sarcoma was heterogeneous contrast enhancement.

## Data availability statement

The data analyzed in this study is subject to the following licenses/restrictions: All the information (except data regarding owners) are described in the article. Requests to access these datasets should be directed to alessia.cordella@outlook.com.

## Ethics statement

Ethical review and approval was not required for the animal study because the retrospective study, imaging and cyto/histology was performed only for dogs' benefit and high standard care. Written informed consent was obtained from the owners for the participation of their animals in this study.

## Author contributions

Conception and design and drafting the article: AC. Acquisition of data: AC and ES. Analysis and interpretation of data: AC, ES, and JS. Revising article for intellectual content: ES and JS. All authors contributed to the article and approved the submitted Version.

## Conflict of interest

The authors declare that the research was conducted in the absence of any commercial or financial relationships that could be construed as a potential conflict of interest.

## Publisher's note

All claims expressed in this article are solely those of the authors and do not necessarily represent those of their affiliated organizations, or those of the publisher, the editors and the reviewers. Any product that may be evaluated in this article, or claim that may be made by its manufacturer, is not guaranteed or endorsed by the publisher.
